# Not Just an Aroma Compound: Expanding Perspectives on Diacetyl in Food Systems and Human Health

**DOI:** 10.3390/molecules31040663

**Published:** 2026-02-14

**Authors:** Emília Maria França Lima, Kayque Ordonho Carneiro, Marcos Vinício Alves, Giselle Santos Silva, Vitor Luis Fagundes, Thyago Matheus Wojcik, Julia Arantes Galvao, Kirill Alexandrovich Lubchinsky, Valentina Nikolaevna Khramova, Svetoslav Dimitrov Todorov

**Affiliations:** 1 ProBacLab, Laboratório de Microbiologia de Alimentos, Departamento de Alimentos e Nutrição Experimental, Food Research Center, Faculdade de Ciências Farmacêuticas, Universidade de São Paulo, São Paulo 05508-000, São Paulo, Brazil; emiliamflima@usp.br (E.M.F.L.); kayqueordonho@usp.br (K.O.C.); marcosv3@usp.br (M.V.A.); giselle_santos@usp.br (G.S.S.); vitorluisfagundes@ufpr.br (V.L.F.); 2 Programa de Pós-Graduação em Ciências Veterinárias, Departamento de Medicina Veterinária, Universidade Federal do Paraná, Curitiba 80035-050, Paraná, Brazil; thyago.wojcik013@gmail.com (T.M.W.); julia.galvao@ufpr.br (J.A.G.); 3 Department of Food Production Technology, Volgograd State Technical University, V.I. Lenin Avenue, 28, 400005 Volgograd, Russia; lubkirill@mail.ru (K.A.L.); hramova_vn@mail.ru (V.N.K.); 4 CISAS-Center for Research and Development in Agrifood Systems and Sustainability, Instituto Politécnico de Viana do Castelo, 4900-347 Viana do Castelo, Portugal; 5 Department of General Hygiene, Sechenov First Moscow State Medical University, Trubetskaya Str. 8, Bldg. 2, 119991 Moscow, Russia

**Keywords:** diacetyl, fermented food, food industry, butter aroma, lactic acid bacteria, sensory quality

## Abstract

Diacetyl has been a known key volatile compound for almost one century, a metabolite naturally produced by different microorganisms during fermentation processes, with traditional applications in food products preparations. Since its discovery, diacetyl has been recognized and actively explored regarding its buttery aroma, which is beneficial for a variety of fermented dairy foods. It is primarily synthesized by lactic acid bacteria (LAB) and other microbial groups through citrate metabolism, a pathway that is strain-dependent and strongly influenced by environmental conditions. Moreover, beyond its sensory relevance, diacetyl has attracted increasing scientific attention because of its antimicrobial activity, including synergistic interactions with bacteriocins and other microbial metabolites, which may enhance food preservation and biotechnological strategies. In contrast, its presence merits attention and needs to be carefully monitored in alcoholic beverages such as beer and wine, where excessive accumulation may compromise product quality. Some studies suggested that diacetyl may have negative health influences and presents safety concerns, as inhalation exposure was associated with pulmonary toxicity and occupational diseases, and was even suggested as one of the risk factors in electronic cigarettes. Emerging studies suggest that diacetyl may exhibit pharmacological potential, including antioxidant, antifungal, and even neuroprotective properties, although research is still in early stages and merits deeper scientific evaluation. Considering its dual nature, beneficial and harmful, this review provides an overview of diacetyl’s properties, safety considerations, and promising applications in biotechnology, biomedicine, and fermented food systems, but with a focus on potential industrial and health hazards. In the current review, we have presented evidence for diacetyl’s beneficial properties and discussed its hazards.

## 1. Introduction

The early understanding of diacetyl emerged alongside the development of applied microbiology. Louis Pasteur first associated lactic acid bacteria (LAB) with beer spoilage and the formation of undesirable buttery off-flavors, which stimulated further investigations into the metabolic origin of this compound. Later, Van Niel [1] identified microbially produced diacetyl as the main contributor to butter aroma, and subsequent studies showed that butter starter cultures contained significant amounts of acetoin and diacetyl. Hays [2] and Hays and Riester [3] further reported the production of these compounds by *Leuconostoc* and *Lactobacillus* species in spoiled orange juice, highlighting the dual role of LAB as both beneficial fermentative organisms and spoilage agents [1,2,3,4].

Diacetyl and structurally related compounds (2,3-pentanedione, 2,3-hexanedione, and 2,3-heptanedione) have been approved as flavoring substances for orally consumed foods by the U.S. Food and Drug Administration (FDA) and the Joint Food and Agriculture Organization/World Health Organization (FAO/WHO) Expert Committee on Food Additives (JECFA) since the early 1980s [5], as well as by the Brazilian Health Regulatory Agency (Agência Nacional de Vigilância Sanitária—ANVISA) [6].

Diacetyl is industrially produced synthetically from butanone and fermentatively through various microorganisms (bacteria part of lactobacilli, lactococci and *Bacillus* spp.) using glucose and other carbon sources as substrates [7,8]. This, diacetyl is widely used in food and beverage flavorings. The principal types of flavorings that use diacetyl are dairy flavors, like cheese, milk, and fermented milk, including yogurt [9]. Diacetyl is sometimes an ingredient in so-called brown flavors such as caramel, butterscotch, and coffee flavors [1,8,9]. The diacetyl-containing flavors may be found in microwave popcorn, snack foods, baked goods, and candies. Also, 2,3-pentanedione is manufactured and used as a flavoring agent due to its similarity to the flavor of diacetyl. It is commonly found in tobacco and related products, spices, extracts, colorings, flavoring agents, condiments, and seasonings [8,9]. Furthermore, diacetyl has antimicrobial and antioxidant properties and anti-browning effects, promoting higher postharvest quality and increasing the shelf life of food commodities [10].

Among LAB, *Lactococcus lactis* subsp. *diacetylactis* and different *Leuconostoc* spp. are responsible for diacetyl production in dairy products. These bacterial cultures can metabolize citrates in the presence of fermentable carbohydrates. This highlights the importance of citrate and specific bacteria in the production of compounds that contribute to the characteristic flavor and aroma of dairy products [11,12]. However, various other microorganisms, primarily yeast and various bacterial species can produce both, diacetyl and acetoin, which also has a slightly buttery flavor. Thus, LAB are of greatest industrial interest and have been most widely studied, although yeasts have great potential for producing these aroma compounds, and studies have shown that other microorganisms produce traces of diacetyl [1,12].

Although diacetyl is intentionally applied or occurs naturally, in certain cases its presence affects the sensory characteristics of food. In beer production, diacetyl concentration is a key quality parameter monitored during fermentation and maturation due to its low sensory threshold and association with undesirable buttery off-flavors [13,14]. Additionally, exposure to diacetyl vapors has been associated with the development of respiratory diseases, initially reported among workers in microwave popcorn manufacturing plants [15].

Despite decades of research, diacetyl remains a compound of dual technological relevance, acting both as a desirable flavor molecule in fermented foods and as a spoilage metabolite or safety concern depending on its concentration and production conditions [9]. This duality has driven extensive studies on its biosynthesis, regulation, and industrial applications. However, the available information is often fragmented across microbiology, fermentation technology, and food safety fields. Therefore, this review aims to provide an integrated overview of the historical development, metabolic pathways, microbial producers, technological applications, and challenges associated with diacetyl in food systems.

## 2. Molecular Structure and Physicochemical Properties

**Figure 1:**
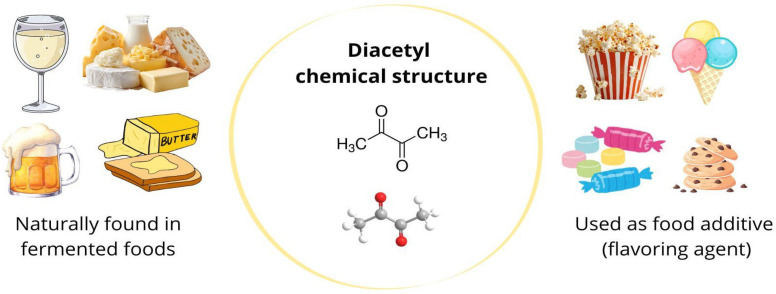
Chemical structure, natural sources, and food industrial uses of diacetyl.

Chemically, diacetyl is characterized by the presence of two adjacent carbonyl groups (–CO–CO–) in a four-carbon backbone (Figure 1). This structure confers high chemical reactivity, particularly toward nucleophilic compounds such as amines and thiols, leading to interactions with proteins and other biomolecules. In the literature, diacetyl is also known as 2,3-butanedione, butanedione, biacetyl, butadiene, and dimethyl diketone, based on different existing biochemical ways of generating names [7,16].

The process of diacetyl production is directly related to carbohydrate and lipid metabolism, a process driven by different microorganisms, but also to the possible consequences of the heat treatment of foods and beverages. The chemical description of diacetyl indicates a relatively low molecular weight, stated as 86.09 M. Moreover, the melting point was determined to be −2 °C, while the boiling point was estimated as 88 °C [7,8]. Further, physicochemical specificity describes diacetyl as a yellow liquid at room temperature and soluble in most organic solvents and water. Considering these facts, it was not surprising that diacetyl presented a high ability to diffuse efficiently in food matrices and biological systems. These physicochemical properties are directly related to their dual role as a desirable flavor compound and a molecule with biological activity [7,8].

Many strains of LAB can produce diacetyl as part of the fermentation of citrate. This compound has antimicrobial activity against yeasts, molds, and Gram-positive and Gram-negative bacteria [17]. The optimization of diacetyl production by LAB starter culture in food processing should lead to biopreservation and the improvement of sensory characteristics of fermented foods [17,18].

**Figure 2:**
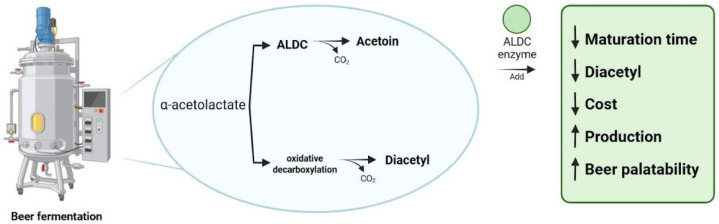
Benefits of using alpha acetolactate decarboxylase in beer production. As a result, maturation time, diacetyl, and cost were reduced (down arrow), while production and beer palatability were improved (up arrow).

The food industry is actively exploring the sensorial and bioprotective properties of diacetyl, which is a widely applied additive due to its flavoring characteristics and its ability to impart a buttery flavor and aroma to foods [19]. Moreover, the application of diacetyl individually or in combination with other flavorings allows for the formulation of foods with distinct flavors and aromas (yogurt, butter, fruit, caramel, vanilla, and others), which can be found in foods such as microwave popcorn, cake mixes, candies, margarines, dairy drinks, ice cream, and snacks. Furthermore, it is naturally found in fermented foods because of their fermentation process [16,17] (Figure 2).

## 3. Main Microorganisms Responsible for Production of Diacetyl

Initially, diacetyl was identified as a metabolite associated with the metabolism of different LAB [1]. As with other microbial metabolites, its production during fermentation is influenced by multiple biogenic and environmental factors, including strain-specific genetic traits and cultivation conditions such as temperature, pH, citrate availability, and cell physiology. Differences between planktonic and immobilized cells have also been reported to affect diacetyl formation during fermentation processes [1,17,20].

LAB are considered a group of microorganisms with several beneficial properties; however, among them, *Lactococcus lactis* is one of the most studied and has been identified as an effective producer of diacetyl. Regarding taxonomic position, *L. lactis* is classified as a species with mesophilic behavior, part of the *Streptococcaceae* family. The genetic diversity of representatives from the species further justified taxonomical division into four subspecies: *L. lactis* subsp. *hordniae*, *L. lactis* subsp. *tructae*, *L. lactis* subsp. *cremoris*, and *L. lactis* subsp. *lactis* (which includes the biological variant diacetylactis) [21]. It is relevant to point out that strains belonging to the *L. lactis* subsp. *lactis* var. *diacetylactis* are described as deficient regarding α-acetolactate decarboxylase, and as a result, they lack acetoin production; however, this is the reason for the significant accumulation of α-acetolactate in the culture medium. However, further spontaneous oxidative decarboxylation of α-acetolactate can lead to excess formation of diacetyl [21]. Such a deficiency in α-acetolactate decarboxylase is in fact what makes these strains excellent candidates for application in butter manufacturing processes and for the production of flavor additives with high α-acetolactate or diacetyl content [22]. Some of the examples are summarized in Table 1, representing different strains of LAB that can produce diacetyl depending on the environmental conditions.

In the evaluation and further quantification of diacetyl production, a colorimetric assay is a common analytical approach. The method was based on the overnight growth of investigated strains, cultured at optimal temperature for 24 h in sterile skimmed milk. Further, the detection of diacetyl was recorded by exposing an aliquot of the fermented milk to α-naphthol and creatine–KOH solutions, where the formation of a pink color band at the liquid surface served as evidence of the presence of diacetyl [23,24]. This qualitative assay has been widely employed to screen diacetyl-producing LAB strains, particularly in dairy substrates. One example is the report of Hosken et al. [17], who evaluated the potential of LAB present in Brazilian artisanal cheeses, including the production of diacetyl. Interestingly, they observed that the occurrence of diacetyl-producing LAB may vary in cheeses depending on the type of endogenous fermentation used in the cheesemaking process. Table 2 summarizes studies that have also reported diacetyl production in different LAB species.

Diacetyl is generally associated with its flavor, either beneficial or indicating spoilage, strictly depending on where this metabolite was produced. In addition, diacetyl and its producers are also recognized regarding their antioxidant properties. It needs to be underlined that antioxidant capacity is generally related to the production of extracellular polysaccharides (EPSs) by LAB, but the role of diacetyl was also investigated [25,26].

In the context of food commodities, the antioxidant properties mentioned play an essential role in the processes improving the stability of fermented products, restricting processes of lipid and protein oxidation during processing and storage. It is important to mention that antioxidants of microbial origin present additional advantages compared to synthetic alternatives, which have some benefits such as fact that they are generally non-toxic, non-carcinogenic, and biodegradable, aligning with the current demand for natural and sustainable ingredients [25,26,27].

Although several synthetic antioxidants are approved for food applications within regulated limits, some compounds, such as Butylated Hydroxyanisole (BHA), Butylated Hydroxytoluene (BHT), and Tert-Butylhydroquinone (TBHQ), have raised toxicological concerns at high concentrations or under prolonged exposure, reinforcing the interest in natural and microbially derived antioxidant sources [28].

**Table 2:** Methodologies reported for the detection of diacetyl production by LAB isolated from food.

Strain	Origin	Result—Diacetyl Production	Reference
*Limosilactobacillus mucosae*	Brazilian goat milk	Qualitative in vitro test: formation of a pinkish ring in tubes.	Moraes et al. [23]
Isolates from dairy products (cheeses and butter)	Ramadi city; Anbar province, Iraq.	Qualitative in vitro test: Voges–Proskauer Test and Brady’s Reagent.	Al-Hashemi et al. [29]
220 LAB isolates belonging to genera *Lactobacillus* *, *Lactococcus*, *Pediococcus.*	Isolates from Brazilian artisanal cheeses	Qualitative colorimetric test of Voges–Proskauer: diacetyl production was indicated by a pinkish ring in tubes.	Margalho et al. [24]
*Weisella paramesenteroides*, and *Weisella cibaria.*	Isolates from Brazilian artisanal cheese	Qualitative in vitro test: formation of a pinkish ring in tubes.	Teixeira et al. [30]
LAB isolates belonging to genera *Enterococcus*, *Streptococcus*, *Lactococcus* and *Leuconostoc*	Artisanal Coalho cheese produced in Pernambuco, Brazil.	Qualitative in vitro test: formation of a pinkish ring in tubes.	Dias et al. [20]
LAB isolates belonging to genera *Lactobacillus*, *Weissella*, *Leuconostoc*, and *Enterococcus*	Pasteurized milk fermented food, and row cow’s milk from Thailand.	Gas–liquid chromatography	Pakdeeto et al. [31]
*Lactiplantibacillus plantarum* L3	Traditional Chinese fermented food	Headspace solid-phase microextraction gas chromatography–mass spectrometry.	Zhu et al. [32]

* Use of term *Lactobacillus* was according to the literature before corrections in taxonomy suggested by Zheng et al. [33].

## 4. Beneficial Properties of Diacetyl

### 4.1. Sensory and Flavoring Applications of Diacetyl in the Food Industry

For more than a century, diacetyl has been recognized as an organic compound, produced by LAB with a beneficial and/or spoilage role in the sensory characteristics of food products. One of the most iconic characteristics of diacetyl is its buttery aroma, an essential organoleptic habitual and highly valued in dairy products, including butter, cheese, fermented milk and yogurt. From the perspective of the dairy industry, diacetyl is considered and commonly regarded as a quality marker, contributing to the natural flavor and creaminess of food [17], as also presented in Table 3. Moreover, dairy-like products or vegan alternatives implement diacetyl-producing strains in fermentation processes with the clear objective of improving sensory properties and providing products with dairy characteristics (buttery aroma) to the consumers, even if the products are considered alternative non-dairy foods. Moreover, for other fermented products, including wine and beer, diacetyl may benefit the complex sensory properties, discreetly contributing to the shape of their sensory specificity profiles. It is a fact that low levels of diacetyl may bring nuances of roasted products like certain types of coffee, in which diacetyl contributes to the aromatic composition in alcoholic and non-alcoholic beverages. Based on almost a century of experience, nowadays diacetyl is widely recognized by the food market as an artificial flavoring agent, most importantly because levels naturally found in foods do not pose a risk to human health [34].

**Table 3:** Reported diacetyl concentrations, application purposes, and functional outcomes in food-related applications.

Beneficial Properties	Diacetyl Concentration	Application Purpose	Observed Effect	Reference
Sensory properties	Diacetyl concentrations ranged between 10^−7^ and 10^−5^ M	Sensory properties in food matrices: dairy products, beer and biscuits	Diacetyl is an important sensory marker to consumers acceptance	Yang et al. [35]
Sensory properties	To improve sensory quality of margarines: 4.5 to 2700 μg/g; for cheeses: 210 to 780 μg/g	Sensory properties of dairy products	Diacetyl improves the sensory quality of dairy products	Rincon-Delgadillo et al. [36]
Antioxidant properties	Treatment with 10 μL/L of diacetyl	Fresh-cut stem lettuce	Effective suppression of enzymatic browning, and extension of shelf life	Li et al. [10]
Antioxidant properties and food preservation	Fumigation treatment: diacetyl water solutions (1, 10, and 50 μL/L)	Prolonging the storage life and quality of apples	Diacetyl improved fruit quality; increased antioxidant capacity	Wang et al. [37]
Antioxidant properties	Diacetyl at 0.4, 0.8, 1.2, 1.6 and 2.0 μL/mL	Antioxidant capacity of diacetyl	Diacetyl was discovered to be an efficient DPPH radical scavenger.	Ling et al. [38]
Antimicrobial activity—antibiofilm	MBIC of diacetyl = 2 mg/mL and MBEC = 4 mg/mL.	Antibiofilm activity against *L. monocytogenes*	Diacetyl reduces the pathogenicity and biofilm formation of *L. monocytogenes.*	Shi et al. [39]
Antimicrobial activity	Diacetyl vapor	Beef meat samples	Reduction in the growth of *S. enterica* (77%) and spoilage microorganisms (93–99%)	Rupérez et al. [40]
Antimicrobial activity—food preservation	Synergism: 1 AU/mL of reuterin with 50–100 mg/kg of diacetyl	Milk	Synergistic action: reduced growth of *E. coli* O157:H7, *S. Enteritidis*, and *L. monocytogenes*.	Langa et al. [41]

It needs to be mentioned that the sensory threshold of diacetyl is extremely low; normally, final concentrations below 0.2 μg/g are sufficient for its perception [36], making it an excellent food additive in the food industry. However, if it is a clearly beneficial characteristic for the dairy industry, then for non-dairy products it may be considered an indicator of poor quality or contamination; simply, a buttery aroma does not fit with all food products. Moreover, some non-dairy products were developed by the food industry with the objective of simulating traditional dairy products. Examples are some vegan products, or margarines. With the objective of improving the sensory properties of margarines, diacetyl can be added to a concentration of 4.5 μg/g and even up to 2700 μg/g, while for some cheeses, levels of diacetyl can be between 210 and 780 μg/g [36]. Rincon-Delgadillo et al. [36] also mentioned that high levels of diacetyl resulted in consumer preferences for low-fat foods. Moreover, in searching for high-diacetyl-producing strains, Zhu et al. [32] reported and evaluated the production of diacetyl by *Lactiplantibacillus plantarum* L3, a strain isolated from a traditional Chinese fermented food. In the fermentation processes, the *L. plantrum* L3 strain resulted in the production of higher levels of diacetyl (0.11303 μg/g) and acetoin (0.03954 μg/g) during yogurt fermentation. In comparison, applying the commercial control strains, only 0.04825 μg/g and 0.02513 μg/g, respectively, of diacetyl and acetoin were produced. As a consequence, the implementation of *L. plantarum* L3 strain as a conjunct starter culture resulted in increased production of diacetyl in fermented milk, leading to a more pleasant aroma, stronger buttery nuances, and even improved creaminess of the final product. Additionally, it is important to mention that the authors reported higher stability of the compound during storage.

With the same objective, in another study, Yang et al. [35] analyzed the presence of diacetyl in different food matrices, including not only dairy products (milk, butter, cream), but also beer and biscuits, confirming its widespread natural occurrence and its essential role as an important sensory marker from the perspective of consumer acceptance. As mentioned in study [35], the diacetyl concentrations ranged between 10^−7^ and 10^−5^ M, values generally considered consistent with consumer acceptance levels in fermented food products. The study by Yang et al. [35] emphasized the fact that the evaluation of the concentration of diacetyl control is essential, since the fact that even small variations, especially in non-dairy food products, can alter the aromatic profile and affect quality perception. Furthermore, the authors pointed out the importance of developing rapid and sensitive detection methods for this compound, such as colorimetric sensors and Surface-Enhanced Raman Spectroscopy (SERS), an analytical approach that can allow for the precise quantification of diacetyl in complex food products. These technological advances are crucial for quality and safety monitoring of foods that naturally contain or generate diacetyl during fermentation [35].

This example clearly shows that new strains with strong diacetyl production capacity can be selected via natural screening, the selection of LAB strains from different fermented food products, and further implementation in innovative foods to naturally enhance diacetyl levels, contributing to sensory quality and reducing the need for synthetic flavorings. However, it is important to underline that the systematic evaluation of the safety and technological properties of new strains needs to be a priority. In addition to its sensory role as a flavoring compound, diacetyl has been investigated for technological applications in the food industry, especially related to its antioxidant activity, as described below.

### 4.2. Antioxidant Properties of Diacetyl

The antioxidant properties of diacetyl are provided by multiple and complementary mechanisms. Some examples can be related to mechanisms involving the synthesis of radical-scavenging metabolites, metal-chelating activities that limit pro-oxidant reactions, and the action of enzymatic systems such as superoxide dismutase, which directly neutralizes reactive oxygen species (ROS) [27,42].

Recent studies have demonstrated that diacetyl exhibits significant antioxidant and anti-browning effects in plant-based foods, contributing to improved postharvest quality and extended shelf life (Table 3).

In the study by Li et al. [10] performed with fresh-cut stem lettuce, treatment with 10 μL/L diacetyl achieved the effective suppression of enzymatic browning, and the authors showed that shelf life can be extended by more than 8 days at 4 °C compared with untreated controls. This effect was primarily attributed to the downregulation of key enzymes in the phenylpropanoid pathway—phenylalanine ammonia-lyase (PAL), cinnamate-4-hydroxylase (C4H), and 4-coumarate-CoA ligase (4CL)—leading to the reduced accumulation of total and individual phenolic compounds. Concurrently, diacetyl enhanced the antioxidant capacity and decreased ROS levels, indirectly reinforcing anti-browning mechanisms [10].

Similarly, in a study conducted by Wang et al. [37] with postharvest apples, diacetyl treatment improved preservation and fruit quality by maintaining total soluble solids (TSS) and titratable acidity (TA), while reducing ROS accumulation through increased antioxidant capacity. Diacetyl also contributed to the structural integrity of cell walls by maintaining cellulose and pectin levels and inhibiting polygalacturonase (PG) activity. Depper analysis and results from performed transcriptomic analysis suggested that diacetyl can actively modulate specific genes associated with ethylene biosynthesis, pectin and starch degradation, cellulose synthesis, and malate transport, all of them known as collectively delaying physiological deterioration during storage [37]. Furthermore, it was suggested by Ling et al. [38] that diacetyl can effectively play a role in scavenging DPPH radicals, which is conducive to its application in preservation and antioxidant properties. DPPH refers to 2,2-diphenyl-1-picrylhydrazyl, a stable free radical widely used to evaluate the radical scavenging and antioxidant capacity of compounds.

Altogether, it can be suggested that diacetyl is a bioactive multitasking compound, capable of modulating antioxidant responses, repressing browning pathways, and maintaining textural and biochemical attributes in fresh produce. More importantly, this supports its potential application in novel postharvest preservation strategies.

### 4.3. Antimicrobial Potential and Synergistic Effects with Bacteriocins and Others Microbial Metabolites

Overall, diacetyl exhibits broad-spectrum antimicrobial activity against pathogenic and spoilage microorganisms through a multifactorial mode of action, including protein denaturation, membrane permeability disruption, metabolic interference, and modulation of virulence- and quorum sensing-related genes (Table 3).

One of the ways in expressing antimicrobial properties is its ability to interact with sulfhydryl (-SH) groups of sulfur-containing amino acids, part of proteins and enzymes. As a consequence, this can lead to the denaturation and loss of catalytic activity and further compromise the microorganisms’ energy metabolism and protein synthesis [41]. In addition, diacetyl can simply penetrate the cell membrane and alter its permeability, leading to ion leakage/disbalance and loss of essential intracellular components, ultimately leading to loss of cell viability [40]. All these can be clearly considered beneficial when antimicrobial properties of diacetyl are targeting pathogenic bacterial species.

Moreover, it was shown that in *Listeria monocytogenes*, diacetyl also interferes with the expression of genes associated with virulence and *quorum sensing*, and was able to reduce adhesion as a consequence of hindering biofilm formation [39]. This is an example, clearly showing that the multifunctional antimicrobial properties of diacetyl are the effect of the combined mode of action, explaining the broad antimicrobial efficacy of diacetyl against pathogenic and spoilage microorganisms, establishing it as a compound of great interest for natural food biopreservation.

By Depper exploring the antimicrobial effects of diacetyl against *L. monocytogenes*, a foodborne pathogen with strong biofilm-forming capacity and resistance to cleaning processes, Shi et al. [39] reported on its strong inhibitory activity. The authors pointed out that diacetyl not only effectively interferes with planktonic cells but also eradicates preformed biofilms. Moreover, in the mentioned study, they determined the Minimum Biofilm Inhibitory Concentration (MBIC) and the Minimum Biofilm Eradication Concentration (MBEC) using diacetyl at 2 mg/mL and 4 mg/mL, respectively. It was stated that diacetyl acts through multiple mechanisms by the inhibition *L. monocytogenes*, including facts that it interferes with the metabolic activity of bacterial cells, reduces cell surface hydrophobicity, thereby hindering adhesion and biofilm formation, and affects the transcription of virulence genes (*inlA*, *inlB*, *mpl*, *hly*, and *prfA*) and *quorum sensing* genes (*agrA* and *agrC*) [39]. Additionally, the authors pointed out that for the destruction of mature biofilms, higher doses of diacetyl were required, and processes mainly causing structural damage to the extracellular polymeric substance (EPS) matrix of the biofilm and reduced biofilm thickness were observed. This example and the mentioned mechanisms reinforce the potential use of diacetyl as a natural biocontrol agent in fresh foods and in surface hygiene applications [39].

It is important to mention that diacetyl antimicrobial efficiency was considerably greater when diacetyl was applied in the gaseous phase compared to the liquid form. In the experimental set-up, for the inhibition of *L. monocytogenes* and *Salmonella* spp. in aqueous solutions, the reported Minimum Inhibitory Concentration (MIC) was around 1024 mg/mL, limiting its use mainly to biofilm control and localized sanitation. However, when applied as a vapor form, the compound achieves even stronger antimicrobial effects at much lower applied concentrations, including against Gram-negative bacteria. All this indicates that diacetyl vapor can be applied in active packaging systems aimed at food preservation and combat versus foodborne spoilage and pathogens. In this context, Rúperez et al. [40] demonstrated that beef samples exposed to diacetyl vapor reduced the growth of *Salmonella enterica* by 77%, while inhibiting by 93–99% some spoilage microorganisms such as *Pseudomonas* spp.

An additional topic that merits attention is the fact that diacetyl acts in synergy with other antimicrobials produced by LAB. An example in this regard is the report of Langa et al. [41], where the combination of diacetyl with reuterin, a broad-spectrum compound produced by some strains of *Limosilactobacillus reuteri*, was investigated, and evidence for synergistic interactions was provided. In the experimental set-up, the combination of 1 AU/mL of reuterin with 50–100 mg/kg of diacetyl resulted in the extension of the lag phase and significantly reduced growth rates of *Escherichia coli* O157:H7, *Salmonella enteritidis*, and *L*. *monocytogenes*, especially under acidic conditions (pH 5.0). Moreover, when applied in acidified milk, the synergistic action between diacetyl and reuterin resulted in reductions of up to 2 log CFU/mL compared to individual treatments, demonstrating potential for direct application in fermented dairy products.

Normally, synergistic interactions between different antimicrobial compounds are consequences of distinct mechanisms for each individual antimicrobial, and in the current scenario, diacetyl inhibits arginine metabolism and disrupts protein integrity, while reuterin reacts with sulfhydryl groups and induces oxidative stress in target cells. As a consequence, their combined application allows for lower effective concentrations of each individual antimicrobial agent and broadens the antimicrobial spectrum, representing a natural and efficient strategy for the biopreservation of fermented and minimally processed foods [41].

Table 3 provides an overview of representative studies describing the beneficial properties of diacetyl in food systems, including sensory, antioxidant, and antimicrobial applications.

## 5. Diacetyl in Alcoholic Beverages and the Importance of Monitoring Its Levels

### 5.1. Presence of Diacetyl in Beers

One of the dogmas of Paracelsus regarding pharmaceutical sciences is the fact that poison depends on the dose. The same can be clearly said for diacetyl and its application in different food products. In fermented alcoholic beverages, acceptable diacetyl levels vary widely according to product type and desired sensory profile, since a buttery flavor is not favorable for all types of food products. Initially observed by Luis Pasteur, and later explored on different occasions, in beer production, the concentrations of cumulated diacetyl are parameter monitoring during the fermentation and maturation process [43]. The principal point is the fact that diacetyl is easily perceptible, conferring an unpleasant buttery flavor to the beer [44,45,46]. Thus, diacetyl concentrations are used as factors determining the quality of the beer, and normally, levels higher than 0.1 mg/L are considered a spoilage factor for quality [13].

This is even more relevant for the lager type of beer, already known as more sophisticated with a milder and cleaner flavor. In these kinds of beers, even lower levels of diacetyl can result in unwanted buttery notes and can result in rejection by consumers. In such cases, concentrations of diacetyl need to remain strictly below 0.1 mg/L, close to the compound’s sensory threshold [14,44]. On occasions when diacetyl exceeds the mentioned limit, the produced beer acquires excessively buttery notes, clearly indicated by specialists as a sensory defect, especially in styles such as Pilsner and Helles. On the other hand, in different types of beers, characterized by more intense and malt-forward flavor, such as Stouts, Porters, and English Ales, small levels of diacetyl can be tolerated to some extent, or even described as desirable, even reaching around 1.0 mg/L, since in these cases diacetyl can be associated with toffee and caramel-like nuances that complement the malt profile [44]. However, clearly the balance between the presence and excess of diacetyl is a key sensory point in beer production, mainly influenced by the yeast strain, the fermentation and maturation conditions, and the “consensus” between producers and consumer preferences.

As brewing is a fermentation process conducted by the combination of different microorganisms, diacetyl concentration can vary significantly depending on fermentation intensity and substrate availability. During beer fermentation, α-acetolactate formed from amino acid metabolism is converted into diacetyl, which may accumulate in early stages of the process. During maturation, yeast reabsorbs diacetyl and reduces it to acetoin and 2,3-butanediol, resulting in a progressive decrease in buttery off-flavors. This mechanism explains the well-known reduction in diacetyl levels as beer ages [43,47]. Pires et al. [14] explored these topics and indicated that in continuous fermentation systems, diacetyl levels in “green beer” on some occasions can even exceed 1.0 mg/L. However, additional adequate maturation steps will be essential in the technological attempt to reduce the levels of diacetyl below the sensory threshold (0.1 mg/L for lagers). Pires et al. [14] showed that levels of diacetyl can be gradually reduced during maturation processes, where an essential role is granted to the yeast, bio-transforming diacetyl. It is important to underline that diacetyl control depends on several factors, including strains involved in brewing processes, substrate composition, viability of free amino nitrogen (FAN) content, and oxygenation conditions of the fermentation system. It was suggested that low levels of valine may positively influence the formation of α-acetolactate, following the formation of diacetyl. Thus, this clearly highlights the importance of principle nutritional balance in preventing the undesirable formation of specific flavors during beer fermentation. As some industrial production brewing technological approaches choose to accelerate the production process, including reducing the maturation time, this can result in beers with buttery flavors and low consumer acceptance due to high levels of diacetyl [14,47].

The appropriate selection of yeast strain to produce beer is a crucial factor in the control of diacetyl concentrations. Yeast plays a crucial role in the conditions of the fermentation process but contributes to valine synthesis, and further to the metabolism of the formed diacetyl, and all combined, it can contribute effectively to the sensory quality of the final products [48]. It is known that valine synthesis occurs in the mitochondrial region of *Saccharomyces* spp., through the metabolism of the precursor α-acetolactate, but in a limiting way [49]. Taking this into consideration, during the fermentation and yeast growth process, excess amounts of α-acetolactate are secreted by the cell membrane into the wort [50], and when this overproduced α-acetolactate is decarboxylated, a rise in diacetyl can be recorded [46].

In some cases, records of excess concentrations of diacetyl can be linked to the metabolic activities associated with poor hygienic conditions and contamination by other microorganisms, such as bacteria from the groups of lactobacilli or the genus *Pediococcus* spp., or *Pantoea agglomerans* [49]. Moreover, the levels of diacetyl also need to be controlled while monitoring the levels of precursor compounds responsible for its formation [51], and special attention needs to be given to the fact that the formation of diacetyl in beer occurs through the non-enzymatic chemical oxidative decarboxylation of α-acetolactate present in the wort [46,50]. Therefore, manipulating the levels of this precursor, both during the maturation period and in the storage of the packaged product, is an effective tool in controlling diacetyl levels and improving quality in beer production [52].

Based on this knowledge, different authors report enzymatic applications for reducing diacetyl and its precursors [52,53,54]. The use of Alpha Acetolactate Decarboxylase (ALDC), an enzyme capable of reducing the concentrations of diacetyl and α-acetolactate, causes a reduction in the product maturation period by 30–35% (8 days), positively impacting production costs [55]. The ALDC enzyme prevents diacetyl production by directly participating in its biochemical pathway, converting the α-acetolactate present in the product into acetoin and CO_2_, as shown in Figure 2 [52]. Choi et al. [56] suggested that a 25% reduction in diacetyl levels can be achieved when implementing ALDC in brewing processes. Moreover, Soares [54] reported a reduction in diacetyl levels when using ALDC, and even fully removing diacetyl from the final products after 6 days of maturation. In contrast, applied controls showed that in beer samples without the addition of ALCD, a diacetyl concentration of 5 ppb on the 8th day of maturation was still present. These results demonstrate a 25% reduction in the beer production period. Boniciu and Stoicescu [57] also reported lower levels of diacetyl when the ALDC enzyme was added (27.90 ppb of diacetyl without enzyme and 9.10 ppb with enzyme). Ferreira [51], when analyzing 60 samples of Sagres Branca beer, reports an average of 22.32 ± 8.65 ppb of diacetyl, values that, when added to the standard deviation, exceed the maximum limit of 30 ppb. Furthermore, the same author reports that 42 samples showed values greater than 17 ppb, levels sufficient to be detected by the palate of some people.

In contrast, although there are established thresholds regarding the presence of diacetyl in beer, its influence on taste acceptance can be individualized according to the type of beer, geographical region, and final consumer [34].

### 5.2. Presence of Diacetyl in Wines

Diacetyl is frequently considered the principal “villain” when beer sensorial properties are discussed. However, in wine production, there is a wide divergence associated with diacetyl presence, being regarded as one of the main components responsible for promoting a specific aroma and flavor, especially in those produced through malolactic fermentation [58].

In winemaking, excessive diacetyl can be naturally reduced during aging, especially after malolactic fermentation, through yeast lees contact and microbial metabolism, whereas enzymatic strategies such as ALDC addition—commonly applied in brewing—are not routinely adopted in wine production. It is a well-known fact that the winemaking process involves an essential role of the combination of yeast and LAB, involving the fermentation of grapes and transforming the material primarily into wine. Microorganisms involved in wine fermentation are able to metabolize citrate into lactic acid and diacetyl as final products during fermentation, thereby influencing the aroma and flavor of wines [59,60].

Levels of diacetyl in different types of wines are closely related to the specificity of primary material and fermentation and maturation processes, but when present in high concentrations (above 5–7 mg/L), it is considered an undesirable factor due to an excessively buttery aroma and can even be the reason for complete rejection of final products by consumers [61]. On the other hand, moderate diacetyl levels of 1–4 mg/L, depending on the type of wine, may contribute positively to its production by conferring buttery and caramel organoleptic characteristics [60]. In this context, Martineau et al. [62] point out that the influence of diacetyl in costumers’ perceptions of wines is particular to each type, varying on average from 0.18 mg/L for Chardonnay, to 0.89 mg/L for Pinot Noir, to 2.68 mg/L for Cabernet Sauvignon. This fact clearly shows that the distinct perception limits of diacetyl in each type of wine is specific. One example is the fact that Chardonnay wine (white wine) has a limit 15 times lower than Cabernet Sauvignon (red wine), demonstrating that light white wines tend to have a lower threshold for diacetyl. But in general, it can be stated that acceptance thresholds vary according to wine type: in red wines, levels generally range between 0.1 and 3.0 mg/L, while in full-bodied white wines, such as Chardonnay, concentrations may reach up to 5.0 mg/L, associated with the complexity and smoothness typical of these styles [61,63].

Therefore, like other fermented beverages such as beer, monitoring diacetyl formation during wine fermentation is essential to ensure sensory quality and consumer acceptance. Diacetyl levels are largely influenced by the microbial consortium employed. The selection and screening of LAB strains with low diacetyl-producing potential, combined with the appropriate control of fermentation parameters—including temperature, oxygen availability, citrate concentration, and timing of malolactic fermentation—represent effective strategies to regulate its formation [61]. Additionally, the enzymatic reduction of diacetyl to less flavor-active compounds (e.g., acetoin and 2,3-butanediol) can further minimize excessive buttery notes. In fermentations where malolactic conversion does not occur, diacetyl concentrations rarely exceed 0.2 mg/L, a level generally insufficient to significantly impact aroma perception [63].

## 6. Undesirable Properties and Risks

### 6.1. Toxicity of Diacetyl

As already mentioned, diacetyl is considered safe (GRAS—Generally Recognized as Safe) for human oral consumption (via ingestion) by the FDA and ANVISA [6]. However, although its application is permitted by health agencies, its ability to cause toxicity in humans was documented as early as 1979 [16].

The use of diacetyl as a flavoring agent in food gained attention in 2000, when the development of lung diseases was reported in workers at a microwave popcorn factory in Missouri, USA [64]. The consumption of diacetyl had been known and considered safe by health agencies until then, but little was known about its inhalation [65]. In that same year, 2000, eight workers in a microwave popcorn factory were reported to have fallen ill with respiratory complications (four workers from the rooms where the flavorings were mixed and four from the packaging area), developing severe bronchiolitis obliterans, an extremely aggressive and disabling disease, requiring lung transplantation in most cases [66,67]. In order to investigate the cause responsible for the health damage to the affected workers, researchers from the Division of Respiratory Disease Studies of the National Institute for Occupational Safety and Health (NIOSH) conducted investigations at the factory. NIOSH found high concentrations of volatilized diacetyl and that workers in the microwave popcorn production plants had higher rates of respiratory problems (chronic cough, shortness of breath, asthma, chronic bronchitis, and airway obstruction). A total of 117 workers were evaluated, and a worsening of lung health was observed according to proximity to the buttery flavoring. Employees in the mixing room were exposed to levels of diacetyl 800 times higher than those in administrative sectors. Workers in general had 2.6 times more chronic cough, 2 times higher rates of asthma and chronic bronchitis, and 10.8 times more pulmonary obstruction than expected among non-smoking employees. In this way, it was possible to establish a strong link between diacetyl exposure and respiratory damage in workers [15].

Moreover, the NIOSH team also investigated the appearance of new reports in five of the other six microwave popcorn factories. NIOSH found a prevalence of respiratory symptoms (shortness of breath, chronic cough, and wheezing) and airway obstruction among workers due to exposure to oil tanks and butter flavorings (diacetyl) [68].

Cavalcanti et al. [69] reported the illness of four young workers (24–27 years old) in a butter cookie factory in the state of Pernambuco, Brazil. The workers were directly involved in the production of the cookie dough and had continuous contact with the butter flavorings, which contained diacetyl. Continuous exposure caused symptoms of dyspnea, cough, and nasal irritation, progressing to bronchiolitis with no signs of improvement in the following 4 years. None of the affected workers were smokers.

Exposure to diacetyl has become a concern among workers in industries producing these food commodities [70,71]. Van Rooy et al. [72] reported illness among employees of a diacetyl flavoring production plant. Among the 206 workers exposed, three were investigated for developing respiratory problems. Among the symptoms, employees reported wheezing, fatigue, fever, night sweats, nasal, eye, and skin irritation. All three cases presented pulmonary dysfunction and development of bronchiolitis obliterans syndrome (BOS). Another 10 employees died, but without confirmation of death related to exposure. Kanwal and Kullman [73] reported illness among workers at a popcorn production plant in Ohio (USA), with airway obstruction in 3 of the 12 workers with the highest exposure to the flavoring. Kanwal et al. [74] also reported on new cases in Iowa (USA) regarding workers in popcorn production plants, especially those involved in mixing the flavoring ingredients (including diacetyl) with the food. In the mentioned study, 6 out of 13 workers presented pulmonary dysfunction, most probably associated with exposure to diacetyl. Moreover, Akpinar-Elci et al. [75] described respiratory dysfunction in workers manufacturing microwave popcorn. The affected employees presented the same symptoms reported by other workers exposed to diacetyl in other similar cases. Furthermore, all nine cases analyzed from 1994 to 2003 presented a progressive decrease in pulmonary function associated with expiratory volume, typical air trapping and pulmonary hyperinflation, diagnostic markers monitored in pulmonary diseases. Due to the typical symptoms experienced by workers affected by diacetyl exposure in microwave popcorn production plants, the development of BOS became popularly known as “popcorn lung”.

The toxic effect of volatilized diacetyl was evaluated in detail in controlled experiments in animal models. Morgan et al. [76] reported on C57BL/6 mice, exposed to vaporized diacetyl for different periods and applied concentrations comparable to conditions present in microwave popcorn production factories. Authors have followed the emergence of acute necrotizing rhinitis, erosive or necrotizing laryngitis, and bronchitis symptoms and disease development in experimental animals. It was shown that two animals exposed to a concentration of 400 ppm of diacetyl were found dead during the experiment. Moreover, twelve animals (three exposed to 200 ppm and nine to 400 ppm) were euthanized due to their moribund state. Along with this, debilitation and hypoactivity were clearly stated and observed in experimental animals with >10% body weight loss. Weight loss was also recorded in cases of affected workers in the cases previously reported, in addition to respiratory symptoms [69].

Furthermore, Hubbs et al. [77] reported that exposure of Sprague–Dawley rats to butter-flavored flavorings resulted in damage to the airways of these animals. In the mentioned study, it was observed that exposure to artificial butter flavoring at different diacetyl concentrations (203, 285, 352, 371 ppm) for 6 h could negatively contribute to alterations in pulmonary and nasal cells and/or inflammation. Moreover, two animals died after exposure to the flavoring compound (diacetyl containing). In similar experimental set-ups, Goravanahally et al. [78] investigated the exposure of male Sprague–Dawley rats to diacetyl, and demonstrated that diacetyl can play a role in the increase in substance P, a neuropeptide related to smooth muscle contraction, vasodilation, edema, and hypersecretion. These symptoms are associated with inflammatory diseases of the airways [79].

However, misdiagnosis was common during the period before the harmful effects of diacetyl exposure were discovered, and the respiratory diseases presented in workers in microwave popcorn factories were commonly identified as asthma [64]. The lack of in-depth investigations prevented adequate treatment and the identification of the disease associated with diacetyl exposure [15]. The development of respiratory diseases, skin, eye, and nasal irritation is directly proportional to exposure [80], meaning that workers with longer periods of exposure and proximity to buttery flavorings, whose main component is diacetyl, developed respiratory problems and BOS [73]. The use of personal protective equipment (PPE) and continuous ventilation in production plants are effective methods to prevent the damage caused by diacetyl vapors [15,81].

Although the development of BOS is common in workers with high exposure to vaporized diacetyl, this disease is relatively uncommon in unexposed individuals. BOS consists of inflammation of the airways and bronchiolar fibrosis, with progressive action [64]. Due to the inflammatory state of BOS, followed by a healing process, fibroblast proliferation and collagen deposition occur, leading to partial or complete obliteration of the airway lumen and resulting in airflow limitation [82]. In addition, due to the narrowing of the affected areas, BOS makes it difficult for air to return, causing air trapping. This dysfunction causes an increase in lung volume, called hyperinflation [64].

Even if diacetyl is classified as GRAS, some studies demonstrate its harmful effects on liver function when ingested [83] and its cytotoxic profile [84]. Diacetyl has the potential for deregulated acetylation [85], which contributes to an increase in reactive oxygen species (ROS) and dysfunctions of enzymatic and non-enzymatic antioxidants [86].

Salama et al. [83] affirms the toxic profile of diacetyl when applied orally in animals and in THLE2 liver cells. The study indicates that the percentage of viability of THLE2 liver cells was reduced as the concentrations of diacetyl increased. In parallel, it was observed that the presence of diacetyl influenced the cell division of THLE2, inducing a state of arrest in the G0/G1 phases compared to the control group. The same study describes the occurrence of areas of necrosis in the liver tissue of male Swiss Albino mice exposed to diacetyl, strengthening the cytotoxic evidence of diacetyl.

Despite being classified as GRAS for oral consumption, increasing evidence indicates that diacetyl exerts toxic effects through multiple and interconnected molecular and cellular pathways, particularly after inhalation exposure [77]. Mechanistically, diacetyl first interacts with the airway epithelium, disrupting epithelial integrity and altering membrane bioelectrical properties, which compromises barrier function [76]. At the molecular level, its highly reactive α-dicarbonyl structure promotes protein modification, deregulated acetylation processes, and the generation of excessive reactive oxygen species (ROS), resulting in oxidative stress and the depletion of endogenous antioxidant defenses, including GSH, SOD, CAT, and GPx [86,87]. This redox imbalance triggers inflammatory signaling, increased cytokine release, and recruitment of immune cells, further aggravating tissue injury [86,87,88,89].

At the cellular level, diacetyl exposure induces cell cycle arrest, apoptosis, and necrosis in epithelial and hepatic cells. Additionally, evidence demonstrates direct interactions with DNA, leading to mutagenicity, genotoxic stress, and increased frequencies of mutant colonies in experimental models [83]. At the tissue and organ levels, persistent epithelial damage and chronic inflammation stimulate fibroblast proliferation and collagen deposition, promoting bronchiolar fibrosis and luminal obstruction, hallmark features of bronchiolitis obliterans syndrome (BOS) [82]. Collectively, these events establish a multilevel toxicity cascade linking molecular oxidative damage to progressive structural and functional impairment of the respiratory system, with potential systemic cytotoxic effects.

### 6.2. Diacetyl in Cigarettes

In addition to its applications in food, diacetyl is also present in flavored tobacco products, where it is used as a flavoring agent to impart sweet and buttery notes. Its incorporation into conventional cigarettes and, more prominently, into electronic cigarettes (vapes), has resulted in a wide variety of appealing flavors, including fruit-, candy-, and dessert-like profiles [66,79]. Although these products are officially marketed for adult consumers, the use of attractive flavor names and marketing strategies that mimic sweets and confectionery raises concerns about their appeal to adolescents and young adults [79,90].

A study by Farsalino et al. [91] verified the presence of diacetyl in 110 of the 159 samples of electronic cigarette cartridges investigated. The average concentration among the samples was 29 μg/mL, observing values three times higher in cartridges with concentrated flavors (these are used for dilution by the consumer). Furthermore, when converting the actual daily exposure values to diacetyl, 52 samples showed exposure levels higher than the limit defined by NIOSH (65 μg/day), with 26 of them at levels 5 times higher than the limit, and one sample reaching a record 490 times higher than the defined limit.

The presence of diacetyl in electronic cigarettes can be found in combination with other flavoring agents. Research conducted by Allen et al. [66] showed that diacetyl can be identified in 39 out of 51 electronic cigarettes, with varying concentrations among the samples. Moreover, among the evaluated 51 e-cigarette samples, the combination of diacetyl with 2,3-pentanedione or acetoin was found in 21 and 38 samples, respectively. Cocktail and brown flavors were found to contain the highest amounts of diacetyl in their composition. Even if some manufacturers among the analyzed samples claimed in their defense that they do not have to use diacetyl in the formulation of their products, the fact is that this compound was recorded. Even if this was a consequence of some chemical interactions in postproduction processes, the fact that e-cigarettes can carry toxic substances does not remove the responsibility from their manufacturers.

Diacetyl presents distinct ways of causing damage to cells, mainly to DNA and the epithelium of the airways [92]. Fedan et al. [93] show that diacetyl has the ability to interact with the tracheal epithelium, affecting its integrity due to its bioelectrical interaction. Furthermore, it was possible to verify that, depending on the concentration of diacetyl, the relaxation and contraction process is affected. One of the mechanisms of the damage caused by diacetyl results in increased sensitivity to methacholine, a cholinergic agonist with bronchoconstrictive action. These findings pointed to the same mechanisms as the symptoms presented by workers exposed to vaporized diacetyl, with affected individuals potentially exhibiting bronchodilation or bronchoconstriction, depending on the intraluminal condition and the level of exposure. Moreover, More et al. [94] give details on the possible interaction of diacetyl with the integrity of cellular DNA. Diacetyl has cancerogenic capacity and is able to modify the composition and structure of DNA, hindering the natural processes of cell maintenance. In addition, minimum concentrations of 50 μM diacetyl were shown to be sufficient to induce apoptosis in SH-SY5Y cells, confirming its mutagenic ability. The mutagenic activity of diacetyl was also reported by Whittaker et al. [95], where there was an increase in the number of mutant colonies of L5178Y/TK^®^ mouse lymphoma cells. Moreover, Bjeldanes & Chew [96] points to the mutagenicity of diacetyl in *Salmonella typhimurium* TA100 strains.

**Figure 3:**
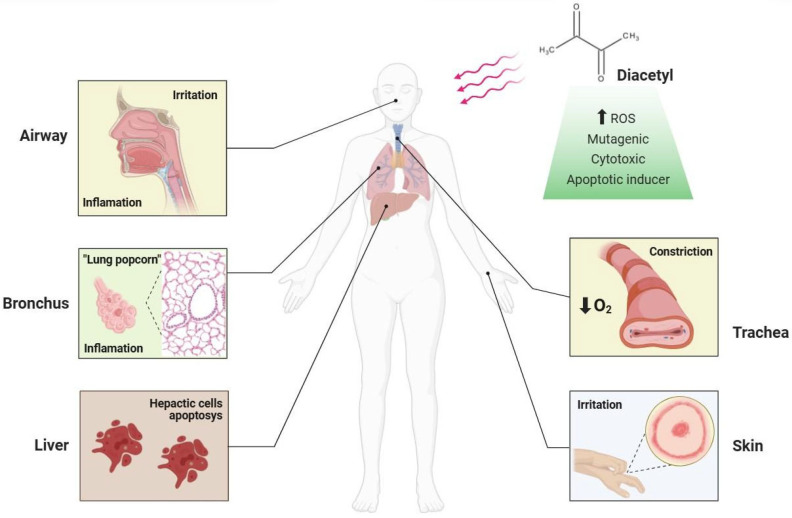
Health impairment associated with high-dose diacetyl exposure, particularly via inhalation. Adverse effects have mainly been reported in occupational settings and are dose- and route-dependent. Oral exposure at levels used in foods is generally regarded as safe.

The identification of diacetyl in other non-food-related products expands the focus on health damage and the development of diseases [90]. Its popular reputation linked to “*popcorn lung*” is a direct warning of its harmful action on the airways and the development of lung diseases [71], in addition to cytotoxic and mutagenic effects [84] (Figure 3). The numerous reports recorded and studied refer to adult workers exposed to vapors of flavorings composed of diacetyl. However, knowledge of damage due to early exposure to diacetyl in adolescents is scarce, and it is speculated that the negative effects are greater, considering the state of psychological, physical, and hormonal maturation of these young people. Importantly, these risks may be amplified in adolescents, who represent a key consumer group of flavored vaping products. The use of e-cigarettes by high school students in the USA has been considered an epidemic due to its alarming increase from 1.5% in 2011 to 27.5% in 2019 [97].

The respiratory system continues to develop throughout adolescence and early adulthood, with ongoing alveolarization, airway remodeling, and maturation of antioxidants and immune defenses. During this period, the airway epithelium is more permeable and biologically reactive to inhaled toxicants. Additionally, adolescents exhibit higher ventilation rates relative to body mass, potentially resulting in greater internal doses of inhaled chemicals [98]. Consequently, exposure to diacetyl during lung development may enhance oxidative injury, disrupt normal tissue maturation, and predispose individuals to persistent airway inflammation, impaired lung growth, and long-term reductions in peak pulmonary function [97]. Early inflammatory or fibrotic changes may therefore have lifelong consequences, increasing susceptibility to chronic respiratory diseases.

Based on the knowledge presented by the scientific community and the reports of illness in workers exposed to diacetyl vapors, should its presence in tobacco products not be reconsidered, given that its inhalation contributes to the emergence of BOS? Does its proven cytotoxic and mutagenic action still classify it as GRAS? The potential for sudden exposure and negative health-related consequences for those working with vaporized diacetyl in food production plants, in addition to the potential cytotoxicity, mutagenicity, and induction of apoptosis based on controlled animal model experiments, direct inhalation through smoking, as well as its application in food, are points that deserve attention and further investigation.

## 7. Emerging Bioactive Properties of Diacetyl and Exploratory Pharmacological Evidence

### 7.1. Recent Studies with Pharmacological or Medicinal Applications

Diacetyl has been well known for almost a century. Initially considered as a beneficial sensorial metabolite, diacetyl has gained attention in pharmacological research. Among its well-known characteristics, its specific antimicrobial activity stands out and is re-evaluated as a potential component in the puzzle of combat *versus* multidrug resistance in human and other animal-related pathogens, which was first described decades ago yet is still widely investigated. Information reported in the last decade showed that diacetyl can inhibit biofilm formation by *L. monocytogenes*, making it a promising compound for controlling this pathogen in foods and contaminated surfaces [39]. Moreover, when incorporated into gelatin-based edible films, diacetyl presented a combination of antimicrobial and antioxidant activities, clearly pointing to its versatility and indicating its potential use as an additive in cheese preservation [99]. Antifungal activity against *Trichoderma lizzi* has also been reported, which may contribute to reducing postharvest losses in plant species [38].

It was shown that diacetyl has also emerged as a potential therapeutic agent, acting as a modulator of amyloid aggregation in neurodegenerative diseases such as Alzheimer’s disease [100]. Its ability to inhibit histone deacetylase suggests possible applications in cancer therapy and neurological disorders, as this enzyme plays a key role in regulating gene expression [101], a fact that merits additional attention and systematic investigations. Thus, although known for a long time, diacetyl has attracted growing interest for its antibacterial, antifungal, antioxidant, and neuroprotective properties, with promising applications in active packaging, food preservation, and pharmacological uses.

### 7.2. Future Perspectives on Diacetyl

Although diacetyl is widely recognized for its sensory properties and, more recently, has been explored for its potential pharmacological activities. This compound also raises important concerns related to toxicity. The fact that diacetyl can be associated with specific toxic effects serves as an alarming indicator that potential dose-dependent negative consequences can be observed, and further investigation into safety needs to be performed.

The scientific community suggests that diacetyl can contribute as a promising pharmaceutical agent with health-promoting applications; however, these investigations remain in early stages, and there is no evidence supporting its safety or efficacy in clinical trials or rigorous in vitro studies. Moreover, several specific concerns regarding diacetyl exposure persist, including the need to develop and validate serological tests capable of identifying biomarkers associated with human exposure to this substance. Despite these concerns, diacetyl is classified by the FDA as GRAS, since its toxicity is primarily associated with inhalation, while it is considered safe when ingested orally [102].

From an industrial perspective, diacetyl represents an important strategic niche within the specialty chemicals and flavor food industries, with a global market value estimated at approximately USD 4.7 million in 2025 and projected to reach around USD 6 million by 2032 [103]. In this context, the controlled microbial production of diacetyl emerges as an alternative approach, enabling the identification and exploitation of microorganisms that naturally synthesize this compound, and even performing fermentation processes with in silico diacetyl production. All this may contribute to new strategies and may promote the development of cleaner and more sustainable processes, reduce environmental impacts, and mitigate health risks associated with inhalation exposure, where food fermentation processes can be rediscovered from the perspective of modern biotechnology. Or, the isolation and application of microorganisms capable of producing metabolites with technological and safety characteristics that will enhance the sensory characteristics of fermented products may meet consumer sensory preferences without relying on genetic modification. This strategy is particularly relevant given the regulatory constraints regarding the use of genetically modified microorganisms in the food industry [104].

## 8. Conclusions

Diacetyl is a long-known microbial metabolite whose relevance extends well beyond its traditional role as a buttery aroma compound in fermented foods. The literature reviewed in this work demonstrates that diacetyl is related to sensory quality, food technology, microbial interactions, and safety considerations. Its production by LAB is strain-dependent and influenced by environmental and technological parameters, highlighting the importance of microbial selection and process control in food systems.

Beyond its sensory contribution, diacetyl exhibits multifunctional technological properties, including antimicrobial, antibiofilm, and antioxidant activities, which have attracted increasing interest in biopreservation strategies. These effects are supported by multiple studies reporting activity against pathogenic and spoilage microorganisms, as well as synergistic interactions with other microbial metabolites. However, the effective concentrations, modes of application, and target systems vary substantially across studies.

At the same time, diacetyl presents well-documented safety concerns, particularly related to inhalation exposure, which have been associated with occupational lung diseases and risks linked to electronic cigarettes. This dual nature underscores the need for a balanced and evidence-based perspective, recognizing that diacetyl is considered safe for oral consumption at regulated levels while posing significant risks under specific exposure routes and conditions.

Despite emerging reports suggesting potential pharmacological or bioactive effects, the field remains at an early and exploratory stage. Future research should focus on clarifying structure–function relationships, standardizing concentration ranges across applications, improving analytical monitoring, and advancing controlled microbial production strategies. Addressing these challenges will be essential to fully harness the technological benefits of diacetyl while minimizing health and safety risks, ultimately contributing to safer, more sustainable, and scientifically grounded applications in food and biotechnology.

## Figures and Tables

**Table 1:** LAB and their metabolites with positive and negative interactions.

Microorganism	Optimum Temperature	Main Metabolites	Secondary Metabolites *
*Streptococcus thermophilus*	40–44 °C	L lactic acid	Acetaldehyde, acetone, acetoin, diacetyl, (ethanol).
*Lactobacillus bulgaricus*	41–44 °C	D lactic acid	Acetaldehyde, acetone, acetoin, diacetyl (ethanol)
*Lactobacillus helveticus*	41–44 °C	DL lactic acid	Acetic acid, acetaldehyde, diacetyl (ethanol)
*Lactococcus lactis*	25–30 °C	L lactic acid	Acetaldehyde, acetone, diacetyl (ethanol)
*Lactococcus cremoris*	25–30 °C	L lactic acid	Acetaldehyde, acetone, diacetyl (ethanol)
*Pediococcus acidilactici*	25–30 °C	DL lactic acid	(Acetoin, diacetyl)
*Bifidobacterium breve*	35–38 °C	L lactic acid, acetic acid	Formic acid, succinic acid, acetaldehyde, acetone, acetoin, diacetyl, (ethanol).

Source: Adapted from Carvalho [1]. * Information in parentheses indicates that these substances are present in very low concentrations.

## Data Availability

No new data were created or analyzed in this study.
